# Effects of physical activity on subjective well-being: the mediating role of social support and self-efficacy

**DOI:** 10.3389/fspor.2024.1362816

**Published:** 2024-05-28

**Authors:** Siqiang Guo, Huaying Fu, Kelei Guo

**Affiliations:** ^1^ School of Physical Education and Health, Zhaoqing University, Zhaoqing, Guangdong, China; ^2^ College of Economics and Management, Zhaoqing University, Zhaoqing, Guangdong, China

**Keywords:** physical activity, social support, self-efficacy, subjective well-being, mediating role, cross-sectional design

## Abstract

**Objective:**

Subjective well-being is an essential component of college students' mental health, and the purpose of this study was to investigate the relationship between physical activity and subjective well-being among college students and to examine the mediating role of social support and self-efficacy between the physical activity and subjective well-being.

**Methods:**

This study utilized a cross-sectional design with a stratified whole group sample of 989 college students (M_age_ = 19.65 years, SD = 1.1) from three universities in Guangdong Province, China, and used the Physical Activity Scale, Subjective Well-Being Scale, Social Support Scale, and Self-Efficacy Scale for data collection. In this study, SPSS 26.0 was used for descriptive statistical analysis and correlation analysis of the collected data. Harman's one-way method was used to test for common method bias.

**Results:**

(1) Physical activity, subjective well-being, social support and self-efficacy were significantly correlated with each other. Among them, physical activity was significantly and positively correlated with subjective well-being (*r =* 0.36), physical activity directly predicted subjective well-being (*β = *0.125, *t = *3.992, *p < *0.01). (2) Physical activity positively predicted social support (*β = *0.386, *t =* 12.505, *p <* 0.01) and self-efficacy (*β =* 0.358, *t = *11.793, *p < *0.01), social support significantly positively predicted subjective well-being (*β = *0.332, *t = *11.370, *p < *0.01) and self-efficacy (*β =* 0.254, *t = *8.744, *p < *0.01), self-efficacy significantly and positively predicted subjective well-being (*β = *0.255, *t = *8.251, *p < *0.01). (3) Not only did social support and self-efficacy play an independent mediating role between physical activity and subjective well-being, but social support and self-efficacy played a chain mediating role between physical activity and subjective well-being.

**Conclusion:**

This study enriched the theoretical guidance for physical activity in promoting college students' subjective well-being. In the practical teaching of promoting college students' subjective well-being, in addition to paying attention to stimulating physical activity, special attention should be paid to the promotion of social support and self-efficacy.

## Introduction

Subjective well-being (SWB) is an important indicator of individual quality of life, which is defined as the individual's overall evaluation of the quality of life at a certain stage according to their own standards, and it has both cognitive and affective components (1). Research findings indicate that over the past decade, Chinese college students have encountered challenges related to academic performance, employment prospects, and economic conditions, resulting in a high prevalence rate of depression reaching 31.38% with an upward trend observed (2). Additionally, emotional stress problems affect up to 47% of college students, while half face sleep issues leading to insufficient rest (3). The sedentary lifestyle characterized by prolonged sitting has become pervasive and is recognized globally as the fourth leading cause of mortality (4), thereby impeding college students' subjective well-being. Consequently, this study aims to explore the impact of physical activity on college students' subjective well-being while investigating whether social support and self-efficacy play mediating roles. The ultimate goal is to provide theoretical guidance for promoting the development of college students' subjective well-being.

### Physical activity on subjective well-being

Physical activity refers to any energy-consuming physical movement resulting from the contraction of skeletal muscles (5). Empirical evidence has demonstrated a positive association between physical activity and subjective well-being (6). Engaging in appropriate physical activities can enhance exercise enjoyment, thereby ameliorating negative emotions such as tension, anxiety, and depression among individuals (7), while also promoting their overall physical and mental health, improving quality of life, and fostering positive emotions that contribute to increased subjective well-being (8). Moreover, both the duration and volume of physical activity influence subjective well-being; as the duration and total amount of exercise increase, so does subjective well-being correspondingly (9, 10). Furthermore, research findings have indicated that interventions involving physical activity can augment an individual's subjective well-being (11). Based on these considerations, we propose Hypothesis 1: Physical activity positively predicts subjective well-being.

### The mediating role of social support

Social support encompasses a range of supportive behaviors from others, which contribute to enhancing individuals' social adaptation and interpersonal skills, thus reducing life stress (12). Not only social support is a significant predictor of mental health (13), but it also plays an essential role in safeguarding the healthy development of individuals (14, 15). Research indicates a substantial positive correlation between the level of social support and the duration of physical activity among college students (16). Furthermore, physical activity positively predicts social support (17–19). Social support serves as a necessary prerequisite for subjective well-being by influencing emotions, cognition, and behavior to promote positive emotions (20) while being negatively associated with negative emotions (21), thus impacting individuals' subjective well-being. High levels of social support facilitate feelings of understanding and respect in individuals and help maintain emotional stability, leading to subjective well-being (22). A robust social support system acts as a buffer against the impact of negative life events (23) and aids in maintaining mental health, recovery capacity, and reducing loneliness as well as the risk for depression (24), ultimately enhancing subjective well-being. Based on these findings, Hypothesis 2 is proposed: Social support positively predicts subjective well-being.

### The mediating role of self-efficacy

Self-efficacy refers to individuals' confidence and belief in dealing with diverse challenges encountered while pursuing specific goals, representing a stable psychological trait (25). Research suggests that participation in regular collective physical activity programs of moderate intensity can enhance self-efficacy among college students (26). Physical activity has a positive influence on self-efficacy, as individuals improve their health and physical abilities through engaging in constructive physical activities (27). Moreover, physical activity facilitates emotion regulation and fosters positive emotions among college students by enhancing personal willpower quality, optimism, and the sense of competence in both academic pursuits and daily life, thereby augmenting self-efficacy levels (28).

Physical activity can mitigate and regulate an individual's negative emotions by attenuating the mechanisms underlying depressive mood (29), thereby bolstering self-efficacy. Self-efficacy is an important psychological construct closely associated with physical activity, and it plays a crucial mediating role in the relationship between physical activity and mental health among college students (30). College students who engage in physical activity demonstrate elevated levels of self-efficacy (31, 32). Moreover, higher self-efficacy among college students is linked to increased social satisfaction and happiness. These individuals possess the ability to manage both positive and negative emotions while fostering optimistic expectations for the future, ultimately enhancing their overall well-being (33). Greater self-efficacy implies heightened trust in one's own abilities and an enhanced sense of control over the environment, which contributes to subjective well-being (34). Conversely, individuals lacking sufficient self-efficacy may encounter challenges such as unclear goals or lack of motivation to achieve them, resulting in diminished subjective well-being (35). Notably, self-efficacy negatively predicts anxiety levels whereby higher levels of self-efficacy are associated with a lower frequency of anxiety symptoms (36). Hypothesis 3 posits that self-efficacy serves as an intermediary factor between physical activity engagement and subjective well-being.

### Chain mediating effect social support and self-efficacy

Previous research has demonstrated a positive correlation between social support and self-efficacy among college students (37). Specifically, it has been found that social support positively predicts college students' self-efficacy levels (38), with parental support being associated with individuals' self-efficacy (39). Moreover, the presence of supportive friends can enhance college students' confidence in coping with adversities and improve their self-efficacy (40). Higher levels of perceived social support encourage individuals to proactively seek assistance from others to overcome challenges, thereby enhancing their self-efficacy. Conversely, insufficient social support may lead college students to adopt maladaptive strategies such as avoidance and self-blame, consequently diminishing their sense of efficacy (41). Additionally, engaging in physical activity has been shown to enhance emotional states such as self-efficacy, belongingness, and achievement while fostering long-lasting subjective well-being (42). Hypothesis 4 is proposed, social support and self-efficacy mediate the relationship between physical activity and subjective well-being.

**Figure 1:**
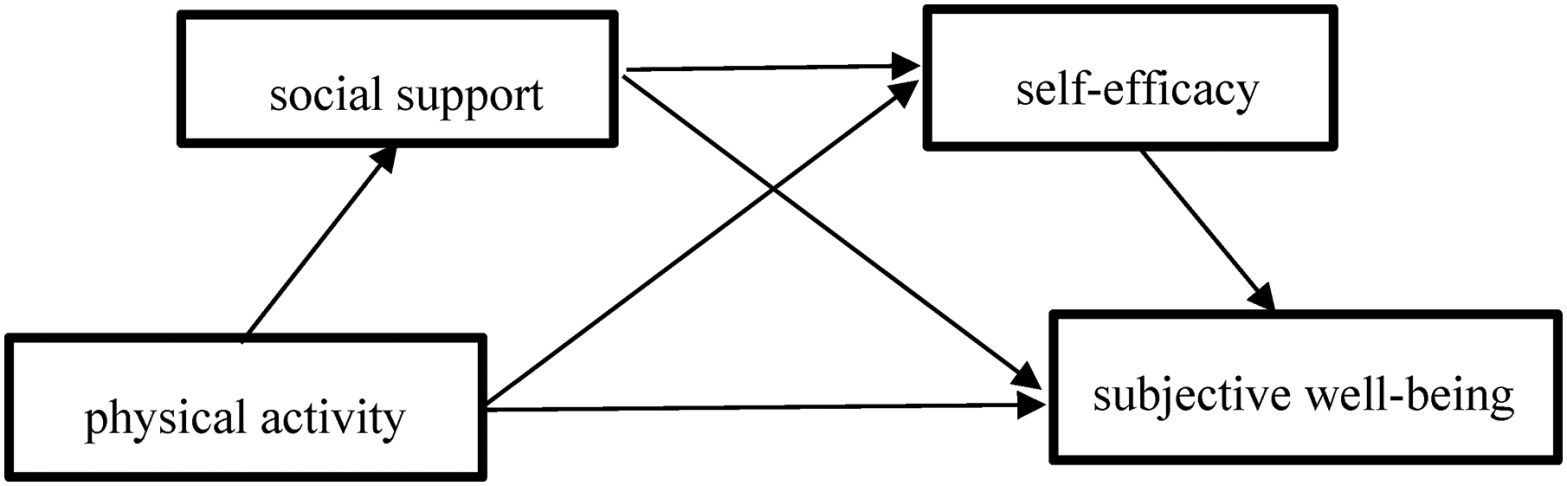
Research on mediation model.

In summary, this study developed a mediation model, as in Figure 1. (1) Examining the positive predictive effect of physical activity on subjective well-being among college students. (2) Examining the mediating role of social support between physical activity and subjective well-being. (3) Investigating the mediating role of self-efficacy between physical activity and Subjective Well-Being. (4) Investigating the chain mediating role of social support and self-efficacy between physical activity and subjective well-being.

## Materials and methods

### Procedure and participants

Using a stratified cluster sampling method, 1,100 participants were selected from Zhaoqing University, Guangdong Technology College, and Guangdong University of Finance in Guangdong Province, the test was carried out by the class unit, with two classes randomly selected from the freshman, sophomore, junior and senior years, for a total of 24 classes. After excluding invalid responses due to regular patterns and missing data, we collected 989 valid questionnaires with an effective recovery rate of 89.9%. We retained valid surveys from 989 students, M_age_ = 19.65 years, SD = 1.1, for analysis. There were 550 (55.6%) men and 439 (44.4%) women; 248 (25.1%) of them were freshmen, 246 (24.9%) sophomores, 247 (24.9%) juniors, and 248 (25.1%) seniors.

### Procedure

In accordance with the Declaration of Helsinki, this study received approval from the Research Ethics Committee of Zhaoqing University (No. 2023-01125-06). The survey was administered through an online questionnaire distributed to the class group by the class director using a secure link. Each questionnaire required no more than 15 min for completion. The emphasis was placed on voluntary participation, data confidentiality, and anonymous responses, while controlling for variables such as gender and grade of participants.

### Measures and instruments

#### Physical activity

The physical activity scale for College Students was adapted from the physical activity commitment intention scale developed by Chen et al. (43) and revised by Wu (44). This scale comprises 8 items with 2 dimensions, where items 1–4 assess commitment to physical activity and are positively scored, while items 5–8 measure adherence to physical activity, with items 5, 7, and 8 being positively scored (e.g., Item 7: “I have the habit of physical activity”) and item 6 negatively scored (e.g., “I have lack of perseverance in physical activity”). Likert 5- point type response format on a five-point scale ranging from “strongly disagree” (1) to “strongly agree” (5) was employed for all responses. The total score reflects the level of physical activity, with higher scores indicating greater levels of physical activity. Previous research has demonstrated the high applicability of this scale among a large sample of Chinese university students (45). In this study, Cronbach's α coefficient for this scale was 0.89.

#### Social support

The social support scale for University Students developed by Ye and Dai (46) was employed, encompassing three dimensions: subjective social support (e.g., Item 1: “Most of my classmates care about me very much”), objective social support (e.g., Item 4: “I often get care and support from my classmates and friends”), and social support utilization (e.g., Item 12: “When I am in trouble, I usually take the initiative to ask for help from others”). A total of 17 items were included in the scale, which utilized a 5-point Likert scale ranging from “not met” (1) to “met” (5). The cumulative score reflects the level of social support experienced by individuals, with higher scores indicating greater levels of physical activity. Previous research has proved the high applicability of this scale among Chinese university students (47). In this study, Cronbach's α coefficient for this scale was found to be 0.94.

#### Self-efficacy

The general self-efficacy scale was developed by Zhang and Schwarzer (48), demonstrating satisfactory reliability and validity. Comprising 10 single-dimensional items (e.g., Item 1: “I can always solve a problem if I try”), the scale employs a Likert 4-point scale ranging from “not at all correct” (1) to “completely correct” (4). The total score reflects an individual's level of self-efficacy, with higher scores indicating greater self-efficacy levels. This scale has exhibited robust reliability and validity among Chinese university students in previous research conducted by Wu et al. (49), In this study, the Cronbach α coefficient of this scale was 0.91.

#### Subjective well-being

The subjective well-being scale comprises two components: the satisfaction with life scale (SWLS) developed by Diener et al. (50), and the positive and negative affect scale (PANAS) developed by Kahneman et al. (51). The SWLS scale consists of 5 single-dimensional items (e.g., Item 7: “pleasant”), rated on a 7-point Likert scale ranging from “strongly disagree” to “strongly agree”, with higher scores indicating greater life satisfaction. The PANAS includes 12 items, wherein items 2, 4, 8, and 10 assess positive emotions (e.g., Item 1: “I’m happy with my life”) while questions 1, 3, 5, 6, 7, 9, 11, and 12 evaluate negative emotions (e.g., Item 10: “impatient”). A Likert's 5-point scale was employed for response options ranging from “almost none” to “very many”. Subjective well-being is calculated as the sum of SWLS score plus PA-NA score; higher scores indicate greater subjective well-being. This scale has demonstrated high reliability and validity among Chinese university students in previous research conducted by Zhu et al. (52). In this study, the Cronbach's α coefficient for this scale was 0.87.

#### Statistical analyses

The data obtained was analyzed using statistical software SPSS26.0. First, the SSPS26.0 program was employed to perform descriptive statistics, including calculating the mean and standard deviation. Second, Pearson Correlation Coefficient was utilized to test the correlation between variables. Third, the macro program PROCESS in SPSS 26.0 was employed to examine the relational models and mediating effects of physical activity, subjective well-being, social support, and self-efficacy. In this study, *p* < 0.05 was set as a statistical result with significance.

## Results

### Common method deviation test

Harman single factor test was conducted to examine potential common methodological biases. Results revealed that there were 8 factors with eigenvalues greater than 1, and the total variance of the first factor was only 25.69%, which fell below the critical standard of 40%. These findings suggest that no significant common method bias exists in this study's data.

### Descriptive statistics and correlation analysis

As shown in Table 1, the correlation coefficients of physical activity, subjective well-being, social support and self-efficacy were statistically significant. Correlation analysis showed that physical activity was positively correlated with social support self-efficacy and subjective well-being. There was no significant correlation between gender and social support. The relationship between variables supports the testing of subsequent hypotheses.

**Table 1:** Means, standard deviations, and correlations among variables.

Variable	M	SD	Physical activity	Social support	Self-efficacy	Subjective well-being
Physical activity	28.60	6.38	1			
Social support	69.72	12.11	0.337**	1		
Self-efficacy	26.61	5.57	0.474**	0.371**	1	
Subjective well-being	22.24	9.64	0.362**	0.476**	0.442**	1

*N* = 989.

** *p* < 0.01.

### Mediating effect test

Based on the correlation analysis, we tested the model for chain mediation effects, and the results are presented in Table 2. Initially, demographic variables such as gender and age were controlled to examine the direct relationship between physical activity and subjective well-being. Prior to introducing mediating variables, a significant direct pathway from physical activity to subjective well-being was observed (*β = *0.362, *t = *12.032, *p < *0.01). Thus, Hypothesis 1 is supported.

**Table 2:** Analysis of regression relationship among variables.

Effect	Item	Effect	SE	*t*	*p*	LLCI	ULCI
Direct effect	Physical activity ⇒ subjective well-being	0.125	0.031	3.992	0.000	0.064	0.187
Indirect effect process	Physical activity ⇒ social support	0.386	0.031	12.505	0.000	0.325	0.447
	Physical activity ⇒ self-efficacy	0.358	0.030	11.793	0.000	0.298	0.417
	Social support ⇒ self-efficacy	0.254	0.029	8.744	0.000	0.197	0.311
	Social support ⇒ subjective well-being	0.332	0.029	11.370	0.000	0.275	0.389
	Self-efficacy ⇒ subjective well-being	0.255	0.031	8.251	0.000	0.194	0.315
Total effect	Physical activity ⇒ subjective well-being	0.369	0.031	12.032	0.000	0.309	0.429

LLCI is the lower 95% limit for Bootstrap sampling and ULCI is the upper 95% limit for Bootstrap sampling.

Subsequently, we conducted a chain mediation analysis to explore the role of social support and self-efficacy in mediating the relationship between physical activity and subjective well-being. As presented in Table 2, the analysis revealed that even after accounting for the mediating variables, there remained a significant direct effect of physical activity on subjective well-being (*β = *0.125, *t = *3.992, *p < *0.01). Furthermore, other direct paths were found to be statistically significant and influenced subjective well-being through three indirect pathways: firstly, physical activity positively predicted social support (*β = *0.396, *t = *12.505, *p < *0.01) which subsequently positively predicted subjective well-being (*β =* 0.332, *t = *11.370, *p* < 0.01), thus confirming hypothesis 2; secondly, physical activity positively predicted self-efficacy (*β = *0.358, *t = *11.793, *p < *0.01) which subsequently positively predicted subjective well-being (*β = *0.255, *t = *8.251, *p < *0.01), supporting hypothesis 3; finally, social support was found to have a positive influence on self-efficacy (*β = *0.254, *t = *8.744, *p < *0.01), validating hypothesis 4.

**Figure 2:**
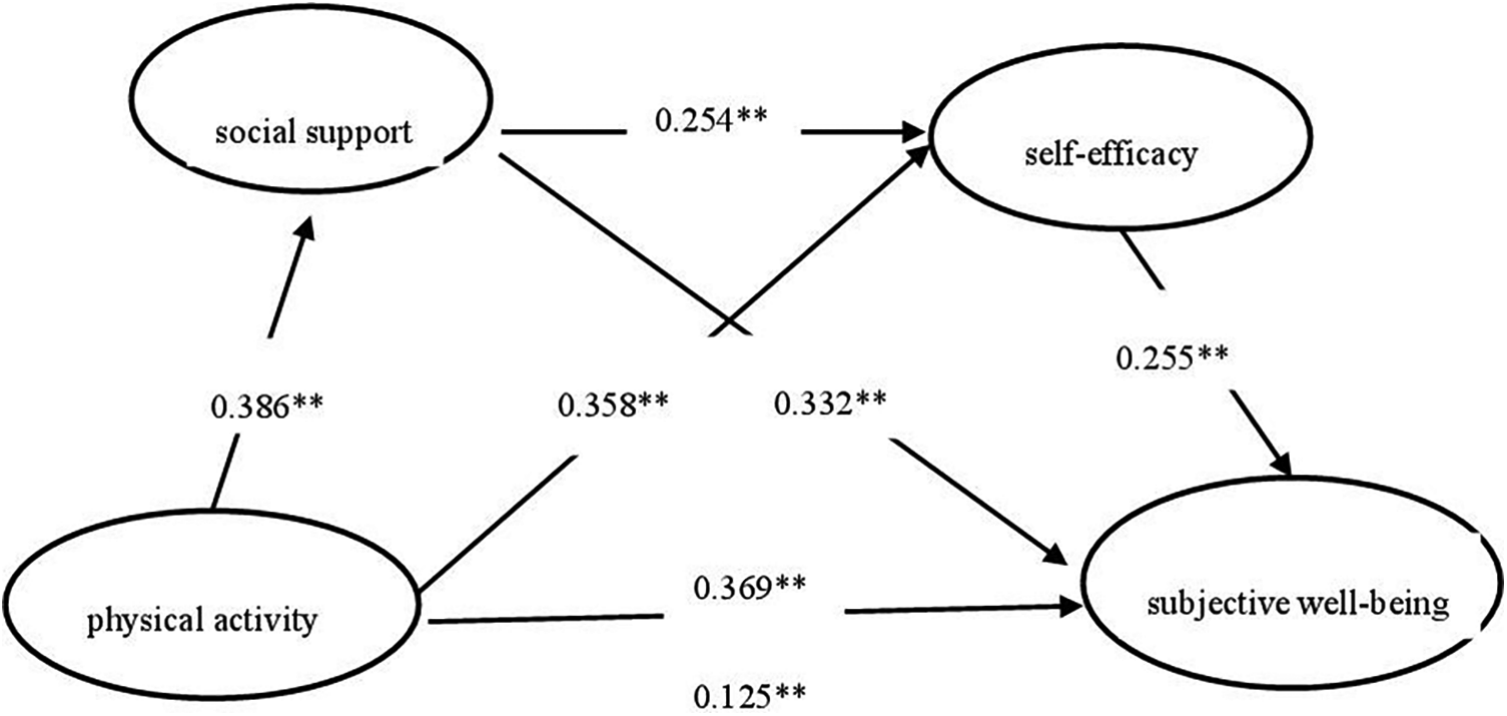
Chain mediation model of social support and self-efficacy between physical activity and subjective well-being.

The deviation-corrected percentile Bootstrap method was employed to conduct the test, with 5,000 repeated samples. According to Table 3 and the path coefficient value shown in Figure 2, the confidence interval results of the mediation bootstrap 95% were obtained as follows: for the pathway from physical activity to social support and then subjective well-being, the confidence interval was [0.979, 0.164], with a mediation effect size of 0.128, the proportion of mediated effects to total effects is 52.5% for the pathway from physical activity to self-efficacy and then subjective well-being, the confidence interval was [0.067, 0.122], with a mediating effect size of 0.091, the proportion of mediated effects to total effects is 37.3%. Finally, for the pathway from physical activity to social support and then self-efficacy before affecting subjective well-being, the confidence interval was [0.016, 0.037], with a mediating effect size of 0.025, the proportion of mediated effects to total effects is 10.2%. The Bootstrap of 95% confidence intervals do not including 0, indicating that the mediating effects are significant, with the mediating effect of social support being the most significant.

**Table 3:** Mediation effect and effect size.

Path	Effect	Proportion of total	95% confidence interval	
			Boot LLCI	Boot ULCI
Physical activity → social support → subjective well-being	0.128	0.128/0.244 = 52.5%	0.979	0.164
Physical activity → self-efficacy → subjective well-being	0.091	0.091/0.244 = 37.3%	0.067	0.122
Physical activity → social support → self-efficacy → subjective well-being	0.025	0.025/0.244 = 10.2%	0.016	0.037
Total effect	0.244		0.020	0.293

## Discussion

### Physical activity and subjective well-being

This study reveals a significant positive correlation between physical activity and subjective well-being, consistent with previous findings (6), thereby confirming Hypothesis H1. In line with the effort-recovery theory (53), college students deplete substantial self-regulation resources when confronted with academic pressure. Consequently, engaging in physical activities becomes essential for stress relief, anxiety reduction, fatigue elimination, and energy restoration to successfully accomplish academic tasks. Positive peer relationships during physical activity can mitigate isolation and enhance overall subjective well-being. Neuroscience research has substantiated that the brain serves as the physiological foundation for happiness generation and subjective well-being perception by orchestrating an integrated circuit of neurotransmitters or hormones such as endorphins, dopamine, and serotonin to regulate emotional responses (54).

### Independent mediating effects of social support

This study found that social support played a mediating role between physical activity and subjective well-being, thereby validating Hypothesis 2, and the results were consistent with previous research.

On one hand, physical activity significantly predicts social support. The goal of engaging in physical activity is to enhance personal subjective well-being and realize untapped potential (55). College students who engage in higher levels of physical activity demonstrate greater autonomous control, enabling them to fully integrate personal development and proactively pursue individual life values. This serves as a strong foundation for acquiring social support. Simultaneously, physical activity fosters self-confidence and enhances social adaptability, facilitating the establishment of mutually beneficial interpersonal relationships and promoting the formation and expansion of social support networks. Research indicates that college students who frequently participate in activities possess a wide range of interpersonal connections and exhibit heightened levels of social cognition, resulting in both increased provision and receipt of material, emotional, and spiritual support compared to those with infrequent or indirect participation.

On other hand, social support exerts a positive predictive influence on the development of subjective well-being. According to the main effect model (56), social support plays a crucial role in maintaining individuals' positive mood and overall physical and mental health when they encounter stress or negative events. High levels of social support are consistently associated with a favorable physical and mental conditions. Establishing robust social support relationships and fostering strong interpersonal connections represent effective strategies for enhancing individuals' social cognition abilities and cooperation skills (57). Moreover, it can effectively alleviate negative emotions such as loneliness and anxiety (58). In essence, social support significantly contributes to promoting greater subjective well-being among college students, which aligns with the findings of this study.

### Independent mediating effects of self-efficacy

This study provides empirical support for Hypothesis 3 by demonstrating that self-efficacy mediates the relationship between physical activity and subjective well-being, aligning with previous research findings indicating a positive association between physical activity and self-efficacy (26), as well as between self-efficacy and subjective well-being (34). By simultaneously examining these three variables, this study highlights the significance of physical activity in fostering the development of both self-efficacy and subjective well-being. Engaging in positive physical activities enables individuals to alleviate negative emotions, thereby promoting an optimistic attitude and enhancing their sense of self-efficacy (59). Moreover, physical activity assists college students in overcoming negative emotions and energy while cultivating self-discipline necessary for scientifically planning study tasks, setting achievable goals, avoiding detrimental temptations or habits, improving academic efficiency, and ultimately strengthening their sense of self-efficacy. Consistent with Bandura's (60) theory on self-efficacy, individuals with higher levels of confidence are more willing to confront challenges head-on and believe in their ability to overcome difficulties through persistent efforts.

In this study, college students' self-efficacy and subjective well-being exert a significant predictive influence. Individuals with elevated levels of self-efficacy demonstrate heightened confidence when confronted with unfamiliar challenges, enabling them to exhibit a positive and assured demeanor in coping with difficulties and setbacks. Simultaneously, during the process of goal attainment and overcoming challenges, their cognitive abilities and perceived control are enhanced, thereby enhancing their self-assessment while inhibiting negative emotions, ultimately fostering an improved state of subjective well-being.

### Chain mediating effect of social support and subjective self-efficacy

Our findings demonstrated that social support and self-efficacy play a mediating role in the relationship between physical activity and subjective well-being, aligning with previous research (37). Therefore, hypothesis 4 is supported. Firstly, social support acts as an external resource closely associated with an individual's intrinsic self-efficacy. Secondly, based on the stress buffering hypothesis of social support proposed (61), it can mitigate the negative impact of stressful events on individuals and reduce their perceived consequences, thereby maintaining positive psychological states and restoring confidence to enhance subjective well-being. Moreover, social support and self-efficacy are interrelated factors that mutually facilitate individual growth. In summary, physical activity predicts subjective well-being by fostering the development of both social support and self-efficacy.

### Practical significance

The significance of this study is to promote subjective well-being among today's college students. This study examines the impact of physical activity on college students' subjective well-being and how it promotes self-efficacy and subjective well-being. Simultaneously, social support plays an important role in promoting self-efficacy and subjective well-being. Firstly, physical activity is recognized as an important antecedent variable of subjective well-being. Therefore, universities should strengthen college students' physical activity. On the one hand, various incentive policies should be formulated to encourage students to participate in more physical activities, and more sports activities and competitions should be organized to provide more opportunities for participation. On the other hand, online publicity and education guidance should be used to make college students correctly recognize the importance of physical activity. Secondly, social support and self-efficacy should be paid attention in improving educators. Universities and families should actively introduce social forces, strengthen internal and external joint construction, and use more social resources to guide college students to engage in social interactions and enhance their confidence in achieving goals.

### Limitations and prospects

This study was limited by time and space factors and utilized a cross-sectional design. Although previous research laid the foundation for this study, it was still difficult to establish a causal relationship between the variables. Future studies may consider utilizing a longitudinal research design. Second, this study used a relatively single research method of questionnaire and mathematical statistics, with sample data coming from individual self-reports. In order to better avoid the problem of common methodological bias, future research could consider the use of multi-subject reporting or other appropriate methods. Once again, this study chose specific colleges and universities as the subjects, which is conducive to internal validity, but also has limitations in terms of external validity. In the future, further validation can be done by focusing on different regions and types of colleges and universities to enhance the credibility of the findings related to this study. Finally, this study only considered the mediating role of social support and self-efficacy in the relationship between physical activity and subjective well-being, however there may actually be other potential mediating variables such as personality, mood, self-esteem and interpersonal relationships. Future studies may incorporate a range of assessment tools and research methods, extend sample sizes, utilize objective evaluation measures, and consider complementary factors to improve our understanding and enhance the subjective well-being among college students.

## Data Availability

The original contributions presented in the study are included in the article/Supplementary Material, further inquiries can be directed to the corresponding author.
